# Pure Motor Monoparesis in the Leg due to a Lateral Medullary Infarction

**DOI:** 10.1155/2012/758482

**Published:** 2012-01-26

**Authors:** Hiromasa Tsuda, Kozue Tanaka, Shuji Kishida

**Affiliations:** Department of Neurology, Tokyo Metropolitan Cancer and Infectious Diseases Center Komagome Hospital, 3-18-22 Honkomagome, Bunkyo-ku, Tokyo 113-8677, Japan

## Abstract

A 76-year-old man with essential hypertension abruptly presented with slight left-sided leg weakness, despite normal strength in the other extremities. Left-sided Babinski's reflex was detected. There were no other neurologic abnormalities. Cranial magnetic resonance imaging demonstrated a small infarction in the lower lateral medulla oblongata on the left side. Cranial magnetic resonance angiography demonstrated an absence of flow of the left vertebral artery. He became asymptomatic within 10 days under intravenous antiplatelet agent. The corticospinal tract fibers innervating the lower extremity caudal to the pyramidal decussation might be involved. We emphasize that this is a first reported case of pure motor monoparesis in the leg due to lateral medullary infarction.

## 1. Introduction

Pure motor monoparesis in the leg (PMML) is characterized by weakness limited to unilateral lower limb without other neurological abnormalities, and caused by isolated corticospinal tract deficits [1, 2]. Regarding responsible infarct lesions for PMML, the cerebral cortex, corona radiata, and internal capsule and pons have been noted [1, 2]. Here, we report a first case of PMML due to lateral medullary infarction (LMI).

## 2. Case Report

**Figure 1:**
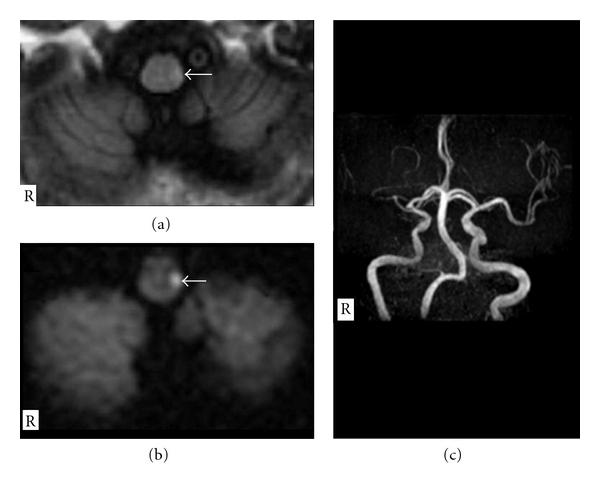
(a) Fluid-attenuated inversion recovery image and (b) diffusion-weighted image of cranial magnetic resonance imaging on the axial images demonstrated a small infarct lesion in the lower lateral medulla oblongata on the left side (arrow). (c) Cranial magnetic resonance angiography demonstrated an absence of flow of the left vertebral artery.

A 76-year-old man with essential hypertension abruptly complained of walking disturbance and was admitted to our hospital on February 2011. In the left lower extremity, Barré's sign was observed, though manual muscle strength testing revealed all normal. In addition, left-sided Babinski's reflex was detected. There were no other abnormalities on the neurological examination. Complete blood cell count and blood chemistry were within normal ranges. Electrocardiogram, echocardiography, and chest X-P findings demonstrated no abnormalities. Cranial magnetic resonance imaging demonstrated a localized LMI on the left side (Figures 1(a) and 1(b)). Cranial magnetic resonance angiography demonstrated an absence of flow of the left vertebral artery (Figure 1(c)). Under intravenous sodium ozagrel at 160 mg/day and the rehabilitation, he became asymptomatic within 10 days.

## 3. Discussion

In LMI, it is well known that various neurological symptoms, such as sensory disturbance, cerebellar ataxia, dizziness, vertigo, nausea, vomiting, Horner's syndrome, skew deviation, ocular tilt reaction, nystagmus, dysarthria, dysphagia, and hiccups commonly occur [3, 4]. On the other hand, Kameda et al. [4] reported that of the 167 patients of LMI, 11 patients (7%) were diagnosed as having Opalski's syndrome, which is defined as LMI case with ipsilateral hemiparesis [4–11]. Opalski's syndrome may be caused by ischemia of the ipsilateral corticospinal tract caudal to the pyramidal decussation [4–11]. Dhamoon et al. [5] described that the regional perfusion failure of the corticospinal tract caudal to the pyramidal decussation might be the result of a hemodynamic mechanism, because this area might be a junctional zone between the anterior and posterior spinal artery and between the vertebral and spinal artery supplied. Whereas, regarding the etiology of Opalski's syndrome, the vertebral artery occlusion [6–8] and infarction of the medullary penetrating artery arising from the vertebral artery or anterior spinal artery [9] were also speculated.

Although the somatotopic arrangements of the corticospinal tract in the human brain have been generally elucidated, very little is known about the somatotopy in the medulla oblongata [12, 13]. In 2011, based on the diffusion tensor tractography analysis, Kwon et al. [13] reported that the hand somatotopy of the corticospinal tract was located at the medial portion of the medullary pyramid, in contrast, the leg somatotopy occupied its lateral portion. However, the somatotopic arrangements of the corticospinal tract in the lower medulla oblongata remain unclear.

To date, there was only one reported case of LMI with ipsilateral monoparesis of the leg [9]. Cranial magnetic resonance imaging demonstrated infarction of the medullary penetrating artery secondary to vertebral artery dissection [9]. Therefore, Liu et al. [9] speculated that the corticospinal tract fibers innervating the lower and upper extremities decussate at different levels. Furthermore, vertigo, nystagmus, Horner's syndrome, ataxia in the upper extremity, and alternating hypalgesia were observed in this case [9]. Whereas, in our present patient, though cranial magnetic resonance angiography demonstrated an absence of flow of the left vertebral artery, cranial magnetic resonance imaging demonstrated that a very small infarct lesion in the lower lateral medulla oblongata on the left side. Therefore, we believed that the corticospinal tract caudal to the pyramidal decussation might be involved by atherothrombotic infarction of the medullary penetrating artery or hemodynamic mechanism due to congenital hypoplasia of the left vertebral artery. Regarding the etiology of unilateral leg weakness due to ipsilateral LMI, we agree with Liu and colleagues' hypothesis [9] that the corticospinal tract fibers innervating the lower and upper extremities decussate at different levels in the lower medulla oblongata. In conclusion, this is a first reported case of PMML due to LMI.
